# LONGITUDINAL METHODS FOR ASSESSING PROGRESSION OF MULTIMORBIDITY AND RELATED HEALTH OUTCOMES

**DOI:** 10.1093/geroni/igad104.0757

**Published:** 2023-12-21

**Authors:** Nicholas Bishop, Corey Nagel, Anda Botoseneanu, Heather Allore, Jason Newsom, David Dorr, Ana Quiñones

**Affiliations:** University of Arizona, Tucson, Arizona, United States; University of Arkansas for Medical Sciences, Little Rock, Arkansas, United States; University of Michigan, Ann Arbor, Michigan, United States; Yale University, New Haven, Connecticut, United States; Portland State University, Portland, Oregon, United States; Oregon Health & Science University, Portland, Oregon, United States; Oregon Health and Science University, Portland, Oregon, United States

## Abstract

The growing body of longitudinal research on multimorbidity (≥ 2 chronic conditions) has identified that multimorbidity progression in mid and late life is associated with change in other health domains (e.g., mobility limitations) and can have far-reaching effects on person-centered outcomes such as health-related quality of life. Several increasingly accessible quantitative methods can be applied to the longitudinal study of multimorbidity and related health outcomes, but there has been relatively little work describing and synthesizing the merits and limitations of methods available for this purpose. We will discuss the application and limitations of popular longitudinal methods that can be applied to the study of change in multimorbidity and associated health outcomes. We will consider overarching issues in the longitudinal study of multimorbidity including operationalizing multimorbidity, data-related concerns such as measurement invariance, and choice of time scale (i.e., change in age or calendar time). We will then cover methods aligned to four specific research objectives: 1) examining individual change in multimorbidity, 2) identifying population sub-groups that follow similar trajectories of multimorbidity progression, 3) understanding when, how, and why individuals/groups shift to more advanced stages of multimorbidity, and 4) examining the co-progression of multimorbidity with key health domains over time. This work will help establish a common language for assessing longitudinal changes in multimorbidity, inform research that can fill gaps in our knowledge regarding multimorbidity progression and critical periods of change, and support the identification of groups that experience divergent rates and etiological pathways of disease progression.

